# Physical Activity Monitoring Using Wearable Devices in Young Adults: Secondary Analysis of a 6-Month Motivational Interviewing Pilot Randomized Controlled Trial

**DOI:** 10.2196/94673

**Published:** 2026-07-16

**Authors:** Nicolette Christodoulakis, Taylor Incze, Sahar Khademioore, Matthew Kwan, Elizabeth Alvarez, Jean-Éric Tarride, Lawrence Mbuagbaw, Laura N Anderson

**Affiliations:** 1 Department of Health Research Methods, Evidence, and Impact, McMaster University, 1280 Main Street West, Hamilton, ON, L8S 4K1, Canada, 1 (905) 525-9140; 2 Department of Child and Youth Studies, Brock University, St. Catharines, ON, Canada; 3 Department of Community Health Sciences, University of Calgary, Calgary, AB, Canada; 4 School of Human Movement and Nutrition Science, The University of Queensland, Brisbane, Queensland, Australia; 5 Centre for Health Economics and Policy Analysis (CHEPA), McMaster University, Hamilton, ON, Canada; 6 Programs for Assessment of Technology in Health (PATH), The Research Institute of St. Joe’s Hamilton, St. Joseph’s Healthcare Hamilton, Hamilton, ON, Canada; 7 Biostatistics Unit, Father Sean O’Sullivan Research Centre, St. Joseph’s Healthcare Hamilton, Hamilton, ON, Canada; 8 Department of Anesthesia, McMaster University, Hamilton, ON, Canada; 9 Department of Pediatrics, McMaster University, Hamilton, ON, Canada; 10 Centre for Development of Best Practices in Health, Yaoundé Central Hospital, Yaoundé, Cameroon; 11 Division of Epidemiology and Biostatistics, Department of Global Health, Stellenbosch University, Cape Town, Western Cape, South Africa

**Keywords:** fitness trackers, exercise, wearable electronic devices, motivational interviewing, pilot study, young adult

## Abstract

In this secondary analysis of a pilot randomized controlled trial of university students aged 18 to 29 years, we assessed the feasibility of providing wearable devices to measure physical activity over 6 months, finding that daily adherence declined over time in both groups, while moderate and vigorous physical activity levels remained similar between the control and intervention groups.

## Introduction

Health behaviors, including physical activity (PA), are important across the life course for the prevention of chronic disease, with young adulthood representing a key period for establishing lifelong health behaviors [1]. PA tends to decline during this transitory period [2], yet few studies have reported device-based assessments of PA in young adults over a long-term period.

We conducted a pilot randomized controlled trial, Motivational Interviewing to Promote Healthy Behaviors for Obesity Prevention in Young Adults (MOTIVATE), to determine the feasibility of a 6-month behavioral and educational motivational interviewing (MI)–based intervention. MI is a person-centered counseling method aimed at enhancing intrinsic motivation for behavior change [3]. This paper examines a secondary aim of the trial by evaluating the feasibility of using wearable devices for monitoring PA throughout the 6-month study period. Feasibility outcomes included adherence to wearing the device and descriptive summaries of activity types.

## Methods

### Overview

In this secondary analysis of a pilot randomized controlled trial, young adults aged 18 to 29 years attending McMaster University were randomized to an intervention or control group [4,5]. Intervention participants received monthly online individual MI sessions from a trained interviewer, along with educational materials. Control participants received educational materials only. The inclusion criteria were a BMI of 18.5 kg/m^2^ or more, access to wireless internet at home, and able to speak English. The exclusion criteria included any physical or mental health conditions that were contraindications to participating in a weight management intervention (eg, eating disorders, pregnancy).

### Ethical Considerations

The trial was registered (ClinicalTrials.gov; NCT05264740), and the Hamilton Integrated Research Ethics Board approved the study (HiREB project 14675). All participants provided written informed consent, data were deidentified, and participants were compensated with CA $30 grocery gift cards for each completed questionnaire.

### Statistical Analysis

All participants received Fitbit Inspire 2 wearable devices to monitor PA throughout the trial. Data were synced to participants’ Fitbit accounts and retrieved by the research team via Fitabase [6], which provides minute-level data collection. Based on prior literature [7], daily adherence was defined as 10 hours or more of wear time per day, determined by heart rate detection. PA intensity was categorized as light (1.5‐3 metabolic equivalents [METs]), moderate (3‐6 METs), and vigorous (≥6 METs) [8]. Weekly adherence was calculated for each participant as a 7-day moving average of daily adherence. Daily hours spent in each intensity category were calculated and averaged. Results are presented by month since randomization and by intervention group. Formal comparisons using statistical tests were not conducted due to limited power and an a priori decision to report secondary outcomes descriptively [4]. Participants also answered two Fitbit-related questions assessing prior experience at baseline and perceived usefulness at the end of the study (Multimedia Appendix 1). Continuous variables were summarized as means (SDs) or medians (IQRs) and categorical variables as counts (percentages).

## Results

Of 120 individuals assessed for eligibility, 101 participants were randomized, and 5 were excluded due to missing Fitabase data (Supplementary Figure 1 in Multimedia Appendix 1), resulting in a final analytic sample of 96 participants (46 control and 50 intervention). The flow diagram for the parent trial is shown in Supplementary Figure 2 in Multimedia Appendix 1.

Baseline characteristics were similar in control and intervention groups (Table 1). Among 96 participants, the mean age was 22.4 (SD 3.6) years, with 38.5% (n=37) identifying as men, 51.0% (n=49) as women, 2.1% (n=2) as genderqueer/nonbinary, and 8.3% (n=8) preferring not to disclose their gender. At baseline, 60.4% (n=58) of participants reported never having used a fitness tracker or health application. By the end of the study, 57.3% (n=55) indicated that using the Fitbit helped them improve their health behaviors (Supplementary Table 1 in Multimedia Appendix 1).

**Table 1.** Baseline characteristics of study population.

Characteristic	Total (N=96)	Control (n=46)	Intervention (n=50)
Age (years), mean (SD)	22.4 (3.6)	23.0 (3.6)	21.8 (3.5)
Gender, n (%)			
Man	37 (38.5)	20 (43.5)	17 (34.0)
Woman	49 (51.0)	24 (52.2)	25 (50.0)
Genderqueer/nonbinary	2 (2.1)	0 (0.0)	2 (4.0)
Prefer not to say or don’t know	8 (8.3)	2 (4.3)	6 (12.0)
Race, n (%)			
South Asian	27 (28.1)	11 (23.9)	16 (32.0)
Middle Eastern	26 (27.1)	17 (37.0)	9 (18.0)
East and Southeast Asian	20 (20.8)	10 (21.7)	10 (20.0)
Black, Latino, Mixed, or another race	10 (10.4)	4 (8.7)	6 (12.0)
White	10 (10.4)	4 (8.7)	6 (12.0)
Prefer not to say	3 (3.1)	0 (0.0)	3 (6.0)
BMI (kg/m^2^), mean (SD)	25.0 (4.9)	25.6 (5.4)	24.4 (4.3)

**Figure 1.**
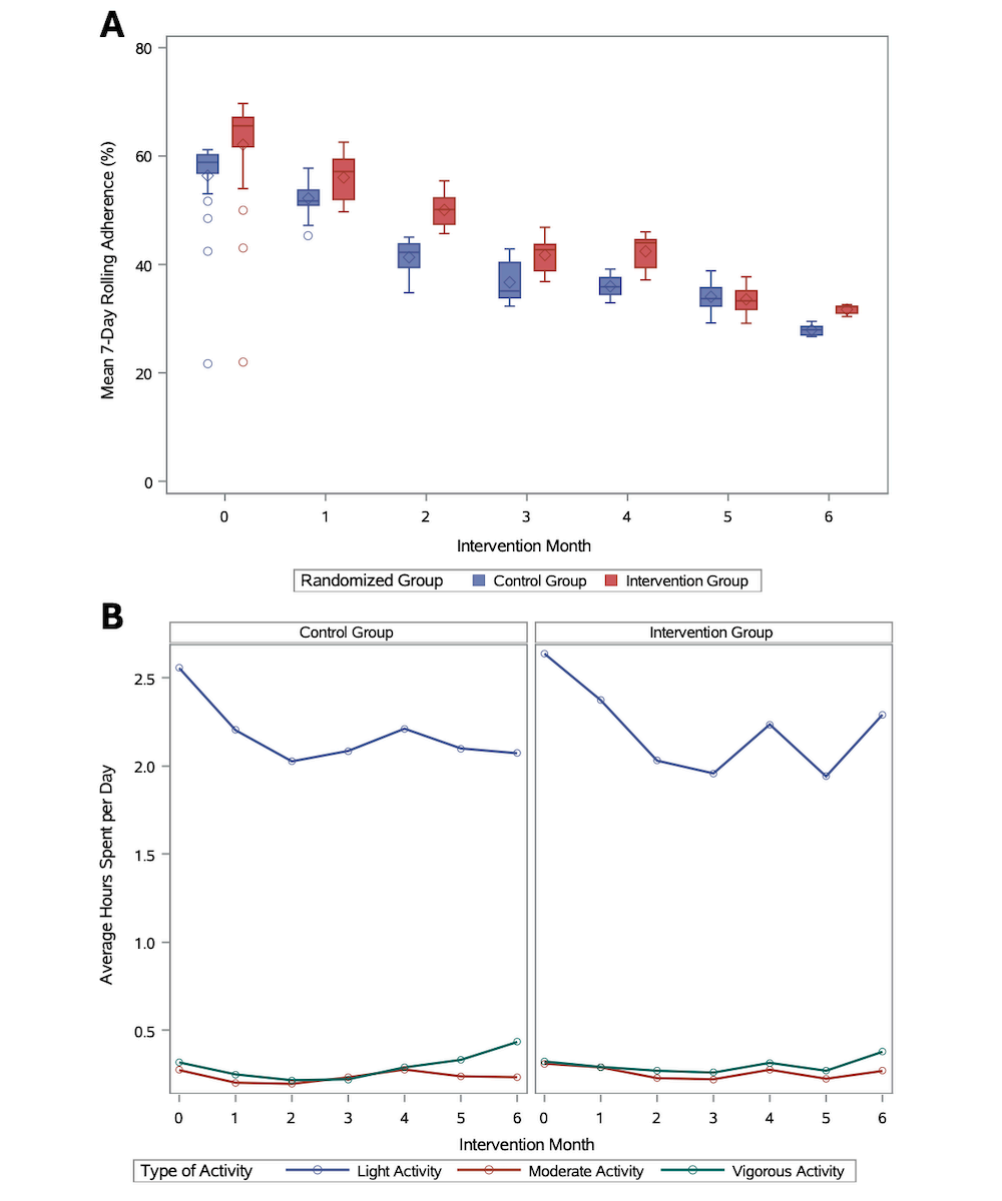
(A) Box plots of the distribution of daily group-level 7-day rolling adherence to wearing the Fitbit, and (B) line plots of average daily hours spent on different types of activity by month since randomization and intervention group.

Most participants (91.7%) had at least one adherent day during the study, including 40 of 46 in the control group and 48 of 50 in the intervention group. The median adherent days were 73.0 (IQR 122.0) for the control group and 80.5 (IQR 121.5) for the intervention group. Adherence declined steadily over the 6-month study period in both groups. The intervention group showed higher adherence than the control group, except during the fifth month, when adherence was similar in both groups (Figure 1A). In terms of PA intensity, there was some variation in light activity, whereas time spent in moderate and vigorous activity remained relatively stable in both groups (Figure 1B). On average, participants spent 2.0 to 2.6 hours per day in light activity, 0.2 to 0.3 hours in moderate activity, and 0.2 to 0.4 hours in vigorous activity.

## Discussion

We observed variability in Fitbit adherence, with the intervention group demonstrating higher adherence than the control group. Adherence declined substantially in both groups over the 6 months, but moderate and vigorous PA levels remained comparable. Additionally, more than half of the participants reported that using the Fitbit helped improve their health behaviors.

These findings are broadly consistent with a previous review among healthy adults that reported that wearable activity trackers were associated with modest increases in PA [9], suggesting that such devices can positively influence behavior, particularly in the short-term. In contrast, a review of children and adolescents found no significant improvements in moderate-to-vigorous activity compared with control groups [10]. This highlights that the effectiveness of wearable devices may vary by population and context. Nonadherence, noncompliance, and reduced engagement with the device over time were common limitations across studies [10], consistent with our observations.

While wearable devices allow continuous monitoring of PA, our results raise questions regarding their long-term feasibility in trial settings. Although moderate and vigorous PA levels appeared stable over time, this may reflect selection bias, as participants with longer follow-up periods were more engaged. Despite gift cards for questionnaire completion (twice per month), adherence declined, suggesting that longer interventions may require additional engagement strategies such as personalized feedback, behavioral prompts, or counseling-based approaches. Given the short duration of previous wearable device studies [10], future trials with longer intervention periods, such as our 6-month design, are needed to assess sustained behavior change. Our findings provide valuable insight into the feasibility of integrating wearables into behavior change interventions. Overall, wearables show clear potential for scalable application in public health, postsecondary education, and primary care settings, where early prevention is crucial.

## Supplementary material

10.2196/94673 Multimedia Appendix 1 Secondary analysis eligibility/randomization flow and participant Fitbit-related responses.

10.2196/94673 Checklist 1 CONSORT-EHEALTH (V 1.6.1) checklist.

## Abbreviations

MET: metabolic equivalent
MI: motivational interviewing
MOTIVATE: Motivational Interviewing to Promote Healthy Behaviors for Obesity Prevention in Young Adults
PA: physical activity
