# Supplementary material for: Physical Activity Monitoring Using Wearable Devices in Young Adults: Secondary Analysis of a 6-Month Motivational Interviewing Pilot Randomized Controlled Trial
Source: JMIR Form Res. 2026 Jul 16;10:e94673. doi: 10.2196/94673 (PMC13375207; doi:10.2196/94673)
Supplement: Multimedia Appendix 1 [file formative-v10-e94673-s001.docx]

**Multimedia Appendix 1**

**Supplementary Figure 1.** Flow diagram of eligibility, randomization, and analysis of participants included in the secondary analysis of the randomized controlled trial.

**Supplementary Figure 2.** Consolidated Standards of Reporting Trials (CONSORT) flow diagram showing participant flow in the parent randomized controlled trial from which the secondary analysis was derived. Figure adapted from Incze et al. [1].

**Supplementary Table 1.** Responses to Fitbit-related questions among participants.

| Question and Response | Total  (N=96) | Control  (n=46) | Intervention  (n=50) |
| --- | --- | --- | --- |
| Prior to the start of this study, did you regularly use any fitness trackers (eg, Fitbit, Apple Watch Fitness) or health apps (eg, MyFitnessTracker, Google Fit, Strava)?^a^ |  |  |  |
| Yes | 38 (39.6%) | 19 (41.3%) | 19 (38.0%) |
| No | 58 (60.4%) | 27 (58.7%) | 31 (62.0%) |
| Do you feel that using the Fitbit helped you to improve your health behaviors?^b^ |  |  |  |
| Strongly Agree | 16 (16.7%) | 8 (17.4%) | 8 (16.0%) |
| Agree | 39 (40.6%) | 18 (39.1%) | 21 (42.0%) |
| Neutral | 16 (16.7%) | 10 (21.7%) | 6 (12.0%) |
| Disagree | 6 (6.3%) | 1 (2.2%) | 5 (10.0%) |
| Strongly Disagree | 2 (2.1%) | 0 (0.0%) | 2 (4.0%) |
| Missing | 17 (17.7%) | 9 (19.6%) | 8 (16.0%) |

^a^ Included in the baseline questionnaire.

^b^ Included in the 6-month (end-of-study) questionnaire.

**References**

1. Incze T, Khademioore S, Alvarez E, et al. Motivational interviewing for health behavior change among young adults in Canada: findings from the MOTIVATE pilot randomized controlled trial. Prev Med Rep. Mar 6, 2026;65:103440. [doi: [10.1016/j.pmedr.2026.103440](https://doi.org/10.1016/j.pmedr.2026.103440)] [Medline: [41846588](http://www.ncbi.nlm.nih.gov/pubmed/41846588)]
